# Cutaneous Manifestations of Anabolic-Androgenic Steroid Use in Bodybuilders and the Dermatologist’s Role in Patient Care

**DOI:** 10.2196/43020

**Published:** 2023-08-03

**Authors:** Garrett Furth, Nathaniel A Marroquin, Jessica Kirk, Hamza Ajmal, Mindy D Szeto, Morgan Zueger, Alyssa P Quinn, Alexa Carboni, Robert P Dellavalle

**Affiliations:** 1 College of Osteopathic Medicine Rocky Vista University Greenwood Village, CO United States; 2 Department of Dermatology University of Colorado Anschutz Medical Campus Aurora, CO United States; 3 Dermatology Service Rocky Mountain Regional VA Medical Center Aurora, CO United States

**Keywords:** anabolic steroids, androgenic steroids, anabolic-androgenic steroids, acne, acne fulminans, isotretinoin, bodybuilding, bodybuilder, social media, skin, dermatology, dermatologist, athlete, sport, steroid, cutaneous

## Abstract

Young adults have been increasingly facing pressure to achieve an appealing athletic physique, often influenced by social media influencers on platforms like Instagram. This viewpoint highlights the association between image-centric social media, dissatisfied body image, the use of anabolic-androgenic steroids (AAS) to achieve desired results, and the overlooked dermatological side effects of AAS, including acne and acne fulminans. We underscore the importance of recognizing acne fulminans as an indicator of possible AAS abuse and encourage dermatologists to actively identify and address AAS use to improve their patients’ well-being.

Physicians should be aware of the increased pressure on young adults to achieve an appealing athletic physique promoted by many influencers on social media platforms such as Instagram. While many claim to have achieved these physiques naturally, some admit to the use of anabolic-androgenic steroids (AAS). Young adults pursuing bodybuilding often use social media as a means of obtaining information for training, nutrition, and supplements [1]. Frequent exposure to these social media images and videos may influence individuals into using AAS to achieve similar results. Image-centric social media is associated with a dissatisfied body image due to physique comparisons to an idealized body, which is also positively associated with AAS use [1]. In the United States, AAS usage is increasing dramatically, reaching up to 80% of amateur bodybuilders and 38% to 58% of weightlifters [2].

While the dangers of AAS are widely known, often overlooked are dermatological side effects including acne, alopecia, striae distensae, hirsutism, and furuncles at injection sites [3]. Acne is a common side effect, reported by 43% of AAS users [3]. AAS are synthetic exogenous testosterone derivatives that result in the enlargement of sebaceous glands, where increased sebum production promotes a suitable environment for the proliferation of *Cutibacterium acnes* [3,4]. Androgen receptors have been found in sebocytes and follicular keratinocytes [3]. The specific AAS mechanism of action depends on the structural variations of each derivative affecting androgen receptor affinity. AAS can increase androgens to supraphysiologic levels, in turn causing increased receptor binding as well as sebum production and *C. acnes* activity, thus risking development of acne vulgaris and acne fulminans (AF) [3].

While rare, AF can be painful and hemorrhagic and is considered the most severe form of acne [2]. The acute development of tender, ulcerative AF nodules on the face, chest, shoulders, and back has been documented by various Instagram AAS users. Systemic symptoms including fever, fatigue, lymphadenopathy, and musculoskeletal pain are possible, as well as an elevated erythrocyte sedimentation rate and leukocytosis [5]. Those who develop AF should be instructed to stop AAS immediately. The treatment of choice for AF is systemic steroids (prednisone 0.5-1 mg/kg/day) and oral isotretinoin (0.1 mg/kg/day). Care should be taken when adding isotretinoin, as it can be a precipitant of AF in the context of AAS use [5]. Initially, steroids should be used alone for 2 to 4 weeks, before adding isotretinoin for 4 weeks. After treatment, the provider should assess the need for continued therapy, steroid tapering, or the addition of other medications.

In a sport where the goal is to build the best physique possible, the effects of AF can be physically and emotionally devastating. The use of AAS and the development of AF, although benign, can lead to permanent scarring, negative self-scrutiny, anxiety, and depression [1]. We hope this viewpoint can highlight this issue while encouraging dermatologists and other care providers to play an active role in recognizing AAS use in patients. Since 50% of AAS users develop acne, AF should be an indicator of possible drug abuse. By recognizing and screening for AAS abuse, dermatologists can help curtail steroid addiction and improve health outcomes for these patients [3].

## Abbreviations

AAS: anabolic-androgenic steroids
AF: acne fulminans
